# The Gulf Coast Health Alliance: Health Risks Related to the Macondo Spill (GC-HARMS) Study: Self-Reported Health Effects

**DOI:** 10.3390/ijerph14111328

**Published:** 2017-10-31

**Authors:** Sharon A. Croisant, Yu-li Lin, Joseph J. Shearer, John Prochaska, Amanda Phillips-Savoy, James Gee, Daniel Jackson, Reynold A. Panettieri, Marilyn Howarth, John Sullivan, Bishop James Black, Joi Tate, Dustin Nguyen, Amber Anthony, Asim Khan, Harshica Fernando, G. A. Shakeel Ansari, Gilbert Rowe, Bret Howrey, Chantele Singleton, Cornelis Elferink

**Affiliations:** 1 Department of Preventive Medicine and Community Health, The University of Texas Medical Branch at Galveston, Galveston, TX 77555, USA; y1ulin@utmb.edu (Y.L.); jjsheare@utmb.edu (J.J.S.); joprocha@utmb.edu (J.P.); jpatstx@gmail.com (J.S.); 2 Sealy Center for Environmental Health and Medicine, The University of Texas Medical Branch at Galveston, Galveston, TX 77555, USA; alanthon@utmb.edu (A.A.); asimhkhan1984@gmail.com (A.K.); csinglet@utmb.edu (C.S.); coelferi@utmb.edu (C.E.); 3 Department of Family Medicine, Louisiana State University at Lafayette, Lafayette, LA 70504, USA; aphil1@lsuhsc.edu; 4 Department of Family Medicine, Lafayette General Hospital, Lafayette, LA 70503, USA; jhgee7@att.net; 5 Center of Excellence in Environmental Toxicology, Perelman School of Medicine, The University of Pennsylvania, Philadelphia, PA 19104, USA; pjacks@upenn.edu (D.J.); howarthm@uphs.upenn.edu (M.H.); 6 Perelman School of Medicine, Rutgers University, the State University of New Jersey, New Brunswick, NJ 08901, USA; rap@mail.med.upenn.edu; 7 Center for Environmental and Economic Justice, Biloxi, MS 39531, USA; Jamb1891@BellSouth.net (B.J.B.); joi830@yahoo.com (J.T.); 8 Mississippi Vietnamese Fishing Community, Gulfport, MS, USA; dustin_ing@yahoo.com; 9 Department of Pathology, The University of Texas Medical Branch at Galveston, Galveston, TX 77555, USA; hafernan@utmb.edu (H.F.); sansari@utmb.edu (G.A.S.A.); 10 Texas A&M University at Galveston; Galveston, TX 77554, USA; roweg@tamug.edu; 11 Department of Family Medicine, The University of Texas Medical Branch at Galveston, Galveston, TX 77555, USA; bthowrey@utmb.edu; 12 Department of Pharmacology and Toxicology, The University of Texas Medical Branch at Galveston, Galveston, TX 77555, USA

**Keywords:** Deepwater Horizon, oil spill, environmental health, petrogenic polycyclic aromatic hydrocarbons (PAHs), Gulf Coast

## Abstract

The Deepwater Horizon (DWH) explosion in 2010 is the largest oil spill (Macondo) in U.S. history. We focused on gaining an understanding of the physical health and mental health effects attributable to the Macondo oil spill. This is a report of a cross-sectional cohort study (wave 1) to establish ‘baseline’ findings and meant to provide descriptive information to be used for a multi-wave, longitudinal study. Gulf Coast Health Alliance: health Risks related to the Macondo Spill (GC-HARMS) uses a Community-Based Participatory Research approach, thus including multi-disciplinary, multi-institutional academic partners and representatives of three communities impacted by the spill. Three research sites were selected for human sampling along the Gulf of Mexico coast including two from Mississippi and one from Louisiana, with Galveston, Texas, serving as a comparison site, given that it was not directly impacted by the spill. One hundred participants were selected from each community, representing adults, seniors and children, with approximately equal numbers of males and females in each group. Participants completed initial assessments including completion of a ‘baseline’ survey and, rigorous physical assessments. Results from wave 1 data collection reported herein reveal changes in self-reported physical health and mental health status following the oil spill, disparities in access to healthcare, and associations between mental health and emotional conditions related to displacement/unemployment. Few environmental health studies have been conducted in communities impacted by significant oil spills. Results imply potential prolonged effects on mental health and community vulnerability.

## 1. Introduction

The largest spill of oil in the history of marine oil drilling operations occurred when the Deepwater Horizon (DWH) oil-drilling rig, operating in the Macondo Prospect (MC252) in the Gulf of Mexico, exploded on 20 April 2010, allowing for more than 4.9 million barrels of crude oil to flow into the ocean unabated from the wellhead for 87 days (~3 months). The well was finally capped on 15 July 2010 [1]; however, researchers discovered evidence of fresh oil in the northern Gulf of Mexico from the Macondo spill 1 year and 10 months (22 months) after it was capped, suggesting that the well continued to leak at least until the end of the study period on 22 May 2012 [2]. Additionally, an estimated 5 million liters of dispersant chemicals were released into the Gulf of Mexico in an attempt to break up the crude oil [3].

The Gulf of Mexico is a rich resource not only for the petrochemical industry, but also for both commercial and recreational fishing. Oil, seafood harvesting, and tourism are all essential to the Gulf’s economy. Oil and gas extraction alone represent about USA $39.8 billion in annual economic activity [4], while in 2013 alone, commercial fishermen operating in the Gulf Region landed 1.4 billion pounds of finfish and shellfish, representing $937 million in revenue [5]. Sport fishing represents a significant portion of dollars spent by residents and tourists to the region. In 2013, over 3.4 million recreational fishermen made over 25 million Gulf fishing trips, of whom, more than 88% were coastal residents. Each of these trips represents a significant impact on local, state, and regional economies due to employment, sales and sales taxes, income, and expenditures on fishing trips and durable equipment. West Florida, for example, attributes 76,000 full- and part-time jobs to recreational fishing activities in the state. Furthermore, in 2013, recreational fishing sales impacts from West Florida contributed $5.3 billion in value added impacts, followed by Louisiana at $1.2 billion. For the same year, Texas sales impacts were estimated at $1 billion, and Alabama’s at $569 million [5]. A major oil spill highlights the potential for conflict between these respective economic drivers—as illustrated by the aftermath of the Macondo spill, which shut down fishing for weeks to months due to concerns about seafood safety [6,7]. In 2012, it was estimated that the DWH disaster over the following short-term seven years (up to 2019) could result in an estimated lost revenue, profit, wages and total economic impact of $3.7, $1.9, $1.2, and $8.7 billion, respectively. This conservative estimate does not include many other impacts on economic losses (e.g., tourism, environmental damage). Long-term losses beyond the aforementioned economic impacts are predicted [8].

Despite the risks posed to human populations by marine petroleum releases, relatively few studies of the impact on human health are available. In their review, Aguilera and colleagues [9] found that of the 38 supertanker accidents in the past fifty years, only seven gave rise to human health studies. More recently, Laffon and colleagues [10] reported that of the 40 post-spill studies identified, only nine examined psychological well-being and human health. Additionally, both reviews found that the majority of research following spills has relied on cross-sectional study designs to assess the association between exposure to oil spills and health outcomes primarily among emergency responders and clean-up workers [9,10]. Studies that have examined mental health following spills have generally reported a spike in acute mental health symptoms. Following the Sea Empress oil spill, researchers found that residents of affected areas were more likely to present with higher anxiety and depression scores, worse mental health and self-reported headaches, sore eyes, and sore throats, all of which were attributed to direct exposure [11]. Similarly, research following the *Prestige* oil spill in Spain and the *Tasman Spirit* spill in Pakistan found reductions in general health and increases in poor mental health and anxiety among affected coastal communities compared to unexposed communities [12,13]. In their assessment of depressive symptoms in communities following the *Exxon Valdez* spill, Palinkas et al. [14] found that the Alaskan Native communities experienced greater psychological effects than Euro-Americans, including increased incidence of post-traumatic stress disorder. They also reported that the Alaska Native communities also tended to have higher exposure scores than Euro-Americans [14]. 

Reports following the Deepwater Horizon spill are similar. In their study of residents of southeastern Louisiana, Osofsky and colleagues found that spill related disruptions in work, family and social life were related to increased symptoms of anxiety, depression and posttraumatic stress [15]. A subsequent report on the same cohort found no significant decline in symptoms at one year [16]. Examining communities in Alabama and Florida, Grattan and colleagues found that directly and indirectly exposed communities showed significant levels of depression and anxiety [17]. They also reported that those with spill-related income loss had significantly worse scores on mental health scales than those with no disruption [17]. The larger Gulf States Population Survey also found that income/job loss was associated with increases in depressive symptoms [18]. Comparing the *Exxon Valdez* and Deepwater Horizon spills, Gill et al. [19] reported that in both cases event-related psychological stress was associated with family health concerns, commercial ties to renewable resources, and about economic future, and economic loss due to exposure to oil from the Deepwater Horizon oil spill. Also related to the Deepwater Horizon oil spill, early findings showed an association between mental health and exposed regions including depression, anxiety and post-traumatic stress disorder (PTSD) [10,15,17,19,20,21,22]. Furthermore, findings from a recent study reported that worry about the impact of the oil spill on health played an intermediate role between physical symptoms and depressive symptoms [23]. Peres et al. [24] examined the physical health outcomes associated with the Deepwater Horizon oil spill that focused on women living in southern Louisiana; they reported an associated increase in self-reported physical health outcomes with strongest associations of burning in the nose, throat, or lungs; sore throat; dizziness; and wheezing. Both direct and indirect exposures to the spill were shown to affect the women’s physical health. 

Literature on longer-term studies is lacking, although recommendations have been suggested to perform medium- and long-term longitudinal epidemiological studies on the human health impacts of exposure to oil spills [10,25]. Following the *Exxon Valdez* spill, commercial fishermen were found to suffer from depression, PTSD, and anxiety for up to six years possibly related to maladaptive coping strategies following economic loss [19,26]. In an analysis one year following the gulf spill, Buttke and colleagues found persistent depressive symptoms and anxiety among affected communities despite declines between 2010 and 2011 [21]. In their study examining anxiety levels at 18 months following the spill, Varner and colleagues report the continued presence of elevated anxiety, particularly in those with direct contact to the oil [27]. 

To give the reader context, The University of Texas Medical Branch at Galveston (UTMB) became involved in the early response to the Macondo/Deepwater Horizon oil spill largely due to ongoing relationships established with coastal communities on various projects in the aftermath of Hurricanes Katrina, Rita, and Ike. A series of communications with over two dozen community groups in the early days of the spill revealed deep concerns over the lack of knowledge regarding the safety of polycyclic aromatic hydrocarbons (PAH) in the oil, its disposition due to the use of dispersants, and persistent uncertainty over their long-term effects on the food web and associated human health effects. This was, and is, clearly magnified by the fact that many coastal families not only subsist on Gulf seafood but also rely on its harvest for financial support. These concerns drove development of a consortium and subsequently, a U19 proposal utilizing a Community-Based Participatory Research (CBPR) approach focused upon gaining an understanding of the long-term health effects attributable to the Macondo/Deepwater Horizon oil spill. The overall goal of this consortium is to conduct a human health assessment of gulf communities that may have been exposed to seafood contaminated with petrogenic PAHs. The Gulf Coast Health Alliance: health Risks related to the Macondo Spill (GC-HARMS) consortium consists of UTMB, the University of Pennsylvania, Texas A&M University at Galveston, Louisiana State University, and Gulf Coast communities impacted by the Deepwater Horizon (DWH) disaster. Consistent with a CBPR approach to scientific investigation, the coalition of community partners has been involved throughout the study, including its planning, implementation, and dissemination of findings. A CBPR approach is an accepted and applicable model to use in post-disaster settings [28,29]. 

Participation sites include the Center for Environmental and Economic Justice (CEEJ; Biloxi, MS, USA), a Mississippi Vietnamese Fishing Community (MVC; Gulfport, MS, USA), the United Houma Nation (UHN; Houma, LA, USA), and Galveston, TX, USA. Seafood sampling and dissemination sites include the Louisiana Environmental Action Network (LEAN, Baton Rouge, LA, USA), the Alabama Fisheries Cooperative (Coden, AL, USA), and Bayou Interfaith Shared Community Organizing (Thibodaux, LA, USA). 

This report of the GC-HARMS consortium provides estimates of the prevalence of both mental and physical health symptoms of selected communities living the Gulf Coast region. This is one of the only studies to date to include both mental health and physical health outcomes associated with the Macondo oil spill in the general community. 

## 2. Materials and Methods

### 2.1. Overall Study Design

This is a report of the first wave (wave 1) of data collection from a multi-wave cohort study of subjects affected by the Macondo oil spill, many of whom are fisher folk and their families. Subjects recruited from the Galveston area serve as a comparison group, given that Texas coastal waters were largely unspoiled by the spill. Each subject was asked to complete a 60-min interview survey about consumption patterns of seafood, exposure to the spill, economic stress and health; to complete a health assessment with a physician; to allow a physician to measure blood pressure, heart rate, height, weight, waist circumference, visual acuity, and lung function; and to provide blood and urine samples. The physician provided the participant with results of the physical and clinical laboratory analysis findings. Code identification numbers were used to protect the confidentiality of the study participants. The study physician was the only person who had access to the participant’s identification information and was additionally bound by physician confidentiality. All other study personnel were blinded in that they could not ascertain the identity of any participant’s questionnaire or study samples. 

The Principal Investigators monitored and evaluated the progress of the study, including periodic assessments of data quality and timeliness, participant recruitment, administration of informed consent, accrual and retention, participant risk versus benefit, and other factors that could affect study outcome, including those that could affect the safety of the participants or the ethics of the study. 

### 2.2. Establishing Cohort

Much work was required in advance to prepare for enrollment of the human cohort, which slowed the ability for rapid data collection. Community coordinators embedded in communities and/or community service organizations were selected, following which, each was required to complete human subjects protection training. We developed and finalized all study protocols, instruments, informed consents (in English, Spanish, and Vietnamese), and received Institutional Review Board approvals (#11-194) from UTMB, the University of Pennsylvania, and Louisiana State University. We established scopes of work for each of the communities, standard operating procedures, and carried out a series of training meetings with local fishers who engaged in seafood sampling. We procured a Certificate of Confidentiality for participants. We also arranged for multi-state licensure of participating physicians who engaged in a rigorous physical examination of subjects that included measurements of blood pressure, heart rate, height, weight, waist circumference, visual acuity, lung function, and blood and urine analysis. Most importantly, we identified local health resources in the event that a subject’s urgent need for medical care was determined as a part of the research process. While this initially delayed our deployment, the work to identify these clinics and in some cases to visit clinic directors in person became a critical factor in seeking urgent care for enrolled participants.

### 2.3. Enrollment 

Three primary research sites were selected to include two communities from Biloxi/Gulfport, Mississippi (MS), including the MS Vietnamese Fishing Community (MVC) and the Center for Environmental and Economic Justice (CEEJ), and one from Southeast LA (UHN). These communities were selected because they bore disproportionate potential risk from long-term consumption of Gulf seafood and/or from specific patterns of consumption, as well as being economically dependent upon seafood harvesting and, thus, at risk of substantial adverse impact due to the spill. While Biloxi and Gulfport are in close geographic proximity to one another, they were both included because the Vietnamese community reported differential consumption and preparation of seafood that may predispose them to greater exposure to contaminated seafood (e.g., by smoking fish whole and consuming the eyes and organ meats). Galveston, TX, USA, was selected to serve as the comparison site as the Texas Coast was largely unaffected by the spill. 

Inclusion criteria were the following: One hundred participants were selected from each community, for a study total of ~400 individuals (300 from Gulf fishing communities and 100 from the comparison community). Because the target communities indicated their interest in including children, adolescents, and elderly in the study, we targeted ~50 adults aged from 20 to 50, 25 adults aged >50, and 25 adolescents and children aged from 5 to 19 from each community, with approximately equal numbers of males and females in each group. Exclusion criteria excluded subjects who were unable to sign informed consent (mentally ill or challenged subjects). Unit of random selection could allow for the selection of up to two adults and two children in a family. Participants were remunerated $50 each for their time. Other benefits included a comprehensive health evaluation, access to laboratory data and health referrals that would promote improved health care. We limited the current analyses to only include adults 18 years and older.

We assisted community hubs with identification of a sampling framework (e.g., the tribal registration for the UHN and the registered members of the CEEJ), from which randomly targeted members were selected for possible inclusion via stratified random sampling to ensure age category representation. We then made multiple visits to each community to publicize the study, to discuss logistics of implementation, potential problems, and to identify clinical sites suitable for carrying out the study. The study coordinator personally trained interviewers and supervised ‘mock’ interviews for training purposes as well as observed ‘real’ study interviews in the field. Trained interviewers administered the survey in face-to-face interviewing sessions. Interviewers input data directly into laptop computers that were secure and encrypted. Once the interview was completed, the interviewer printed a hard copy of the questionnaire and reviewed the results with the participant to ensure accuracy of all entries. Upon completion of the questionnaire, the interviewer affixed corresponding labels to all biological specimen containers. All data were de-identified. The same selection criteria and sampling process, questionnaires, data collection and evaluation techniques were used for the study and reference communities. Baseline data collection for study participants was conducted during the following timeframe: 13 August 2013 through 12 December 2013; and for the comparison community, 27 April 2014 through 2 May 2014. Response rates (i.e., initial plus one or more follow-up assessments) were excellent: (MVC = 100%; CEEJ = 96%; UHN = 91%; Galveston = 99%). The planned length of time between wave data and specimen collections was one year.

### 2.4. Questionnaires

Overall health and well-being was assessed via a 74-item survey given to each respondent. Questionnaires were developed with full input from our community partners and were created for the study using scales of other questionnaires that were validated. Certain questions were unique due to the DWH oil spill. Pilot questionnaires were field-tested using selected representative groups identified by our community partners. The questionnaire was then refined to improve clarity and comprehension within our target population. The final instrument contained multiple descriptive variables that included the following: health symptoms and degree of belief that they are associated with the spill, indicators of general health, respiratory symptoms, chronic health problems, access to medical and mental health resources, mental health (depression, anxiety, PTSD, coping skills and social support), habits and lifestyle, brief family history, occupational and residential histories, fishing, seafood consumption, and trust in social institutions. All study instruments, consents, and recruitment materials were made available in English, Spanish, and Vietnamese. Translations were furnished by professional translators and back-translated by our community partners to ensure accuracy and cultural acceptability.

Anxiety was assessed with the Generalized Anxiety Disorder-7 scale (GAD-7) [30]. This instrument asks the respondent “Over the last two weeks, how often have you been bothered by the following problems?” The problem list includes the following: feeling nervous/anxious, not being able to sleep/control worrying, worrying too much, trouble relaxing, restless/hard to sit still, easily annoyed/irritable, and feeling afraid. The response range for each item (0–14 days) was coded as not at all (0–1 days = 0), several days (2–6 days = 1), more than half days (7–11 days = 2), and nearly every day (12–14 days = 3). The scores for the seven items were then summed (range 0–21) and interpreted as minimal anxiety (0–4), mild anxiety (5–9), moderate anxiety (10–14), and severe anxiety (15–21).

Depressive symptoms were assessed with the eight-item Patient Health Questionnaire depression scale (PHQ-8) [31]. Respondents were asked in the general form “Over the past two weeks, how many days have you …?” Responses were categorized as not at all (0–1 days = 0), several days (2–6 days = 1), more than half days (7–11 days = 2), and nearly every day (12–14 days = 3). The items were then summed (range 0–24). The scores for depressive symptoms were interpreted as none (0–4), mild (5–9), moderate (10–14), moderately severe (15–19), and severe (20–24). 

PTSD was assessed with the Primary Care PTSD screen (PC-PTSD) [32]. The four items in this scale were worded to focus on the oil spill and asked the respondent “During the past 30 days have you…” Specific items addressed nightmares, trying not to think about the spill, being constantly on guard/watchful/easily startled, felt numb or detached. Responses were coded Yes (1) or No (0) and summed to form a 4-point scale. The presence of PTSD was interpreted as a score of 3 or greater.

Resiliency and Coping was assessed with four items adapted from the Self-Mastery Scale [33]. These items assessed the extent to which the respondent believes that life’s chances are under their own control rather than fate. Items were rated on a 5-point scale—strongly disagree to strongly agree (range 1–5) and summed (range 4–20) with higher scores indicating higher levels of self-mastery. The items were as follows: (a) I am in control of my future; (b) I can do just about anything I really set my mind to; (c) I am confident in my ability to handle unexpected problems; and (d) When I need suggestions about how to deal with a personal problem I know there is someone I can turn to.

Social Support was assessed with four items rated on a 5-point scale—never to always (range 1–5). The items were as follows: (a) have you had someone willing to listen to you; (b) have you had contact with people in a similar situation; (c) did you receive practical help (financial, repairs, meals); and (d) I first turn to my family and neighbors for assistance. 

Health status was determined in two ways. First, respondents were asked to rate their general health on a 5-point scale from very good to very poor both prior to and after the oil spill. These responses were dichotomized into good/very good versus fair/poor/very poor. Second, respondents were asked if they had ever been told by a doctor that they had hypertension, diabetes, heart trouble, stroke, or cancer. Each item was coded Yes (1) or No (0).

Access to healthcare was assessed with four items: (1) Do you have any kind of health care coverage? (2) Does your health care plan include mental health coverage? (3) Do you feel you have access to any health care professional to help with and treat your problems? (4) Do you know of a clinic or health care provider where you can go to get medical care? For each community, the proportion of subjects who answered “Yes” to each question was calculated.

Covariates: Other variables included community (CEEJ, MVC, UHN, or Galveston), age at initial interview, gender, ethnicity, participation site, language of interview, current smoking status, body mass index (BMI). BMI was also categorized as overweight (BMI ≥25 and BMI < 30) and obese (BMI ≥ 30). Economic stress was assessed with the question “Has your family’s income been impacted negatively by the spill?” Yes (1) or No (0). Respondents were also asked about changes in employment and changes in living arrangements, Yes (1) or No (0), as a result of the spill. Spill exposure was assessed with the question “Were you exposed directly to the oil spill?” Yes (1) or No (0). 

### 2.5. Data Analysis 

Study subjects from each community were characterized by age, gender, ethnicity and language. The difference among communities in demographics and self-reported conditions was examined by Fisher exact test with Monte Carlo estimation. Differences in self-rated health, presence of PTSD, access to healthcare, seafood quality and seafood consumption were evaluated across sites with χ^2^ and Fisher’s Exact tests. Differences in GAD-7, PHQ-8, and resiliency scores were compared with ANOVA and Tukey tests. 

Because of differences across sites in self-rated health and PTSD, multivariate regression was used to examine the relationship between covariates (demographics, social support, financial strain, employment status, living situation, oil spill exposure, health conditions, and access to healthcare). The binary outcome of good/very good self-rated health was modeled with logistic regression with robust standard errors. Because of the skewed distribution of PTSD across sites, logistic regression models suffered from problems of separation. Thus, PTSD was modeled as a count of positive responses to the PTSD scale questions using negative binomial regression. These models included demographics, social support, financial strain, employment status, living situation, oil spill exposure, health conditions, and access to healthcare. Only variables with statistically significant bivariate relationships with the outcomes were retained in the final models. Since responses to some questions (“don’t know” and “not applicable”) resulted in missing values, multiple imputation with chained equations was used to perform sensitivity analysis of the final models.

Study personnel and statisticians were blinded in that they only had access to de-identified data. All tests were two-sided and *p*-values < 0.05 were considered statistically significant. All analyses were performed with Stata 14 MP (Stata Corp., College Station, TX, USA). 

## 3. Results

### 3.1. Demographics

Largely due to the diligence of our community coordinators, we successfully recruited our targeted population from each of the study sites. As observed in Table 1, age group inclusion is approximately what we had intended. Gender is skewed in one population (CEEJ); however, our community partner indicates that this is representative of the population. Ethnicity is expectedly skewed given the populations chosen for inclusion (*p* < 0.01). It is important to note that differences in language do exist among one population (MVC) with nearly 99% of the population speaking Vietnamese as their primary language.

### 3.2. Perception of Health

**Figure 1:**
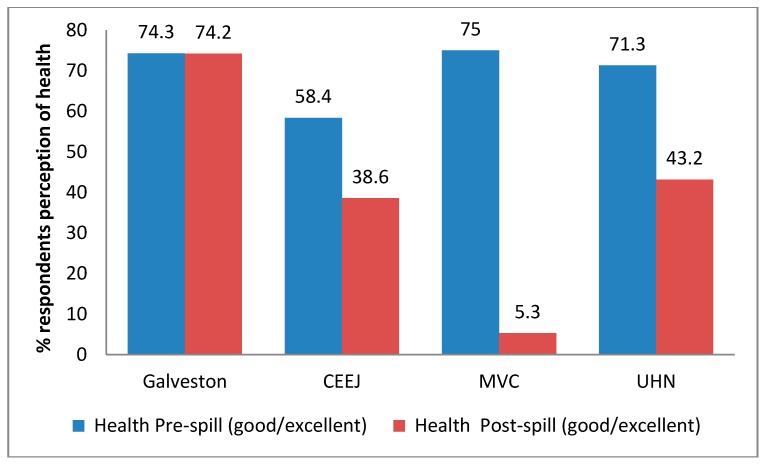
Perception of health pre- and post-spill. The difference in the proportion of respondents reporting “Good” or “Very Good” health among four communities was examined by χ^2^ test. There was no difference among the communities before the spill (*p* = 0.10); however, there was a significant difference after the spill (*p* < 0.001). Center for Environmental and Economic Justice (CEEJ); Mississippi Vietnamese Fishing Community (MVC); United Houma Nation (UHN).

Overall health and well-being was assessed via a baseline questionnaire given to each respondent. Our results indicate that all four groups had more than 60% of respondents rate their health as “Good” or “Very Good” before the spill. However, after the spill, all three study groups directly affected by the spill saw diminished self-reported health status. As observed in Figure 1, comparisons of self-reported health did not statistically significantly differ across sites prior to the spill (*p* = 0.06). However, marked differences (*p* < 0.01) were observed following the spill. These data suggest that those communities near the spill had negative self-reported health status and that some communities’ health statuses were particularly susceptible. 

### 3.3. Self-Reported Health Conditions

Health status was further investigated through the health questionnaire. The percentage reporting each chronic condition across the four communities is summarized in Table 1. The proportion of respondents reporting hypertension was significantly higher in the CEEJ, MVC and UHN communities compared to Galveston. The rate of diabetes was also higher in these communities, ranging from nearly double in CEEJ to nearly three-fold higher in UHN compared to Galveston. Other chronic conditions were similar across sites. 

### 3.4. Mental Health Indicators

Responses to the anxiety scale (GAD7 ≥ 10) and the depressive symptoms scale (PSQ8 ≥ 10) showed some elevation in the UHN community but no statistical difference across groups. Rates of severe anxiety ranged from 1.3% in Galveston to 2.5% in MVC, while rates of severe depression ranged from 1.3% in CEEJ to 6.3% in MVC. The PTSD scale (PC-PTSD ≥ 3) showed large differences in prevalence across groups range from zero (0.0%) in Galveston to 19% in MVC (*p* < 0.001).

### 3.5. Access to Healthcare

**Figure 2:**
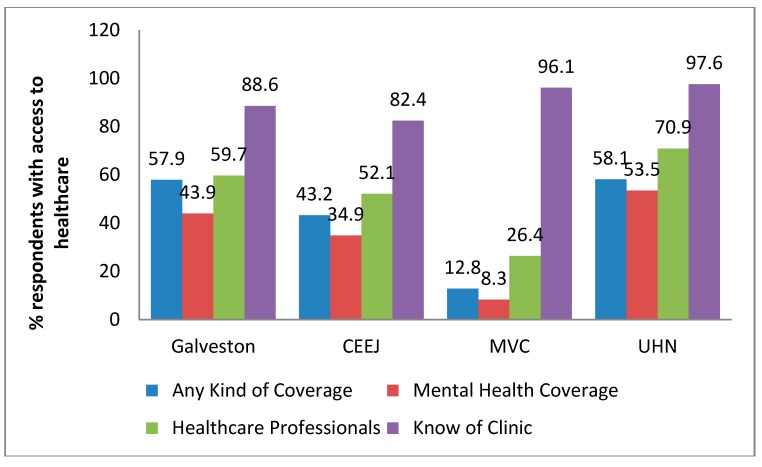
Access to healthcare. Questions related to access to health care coverage and access to a clinic or health care provider reveal disparities across communities.

Each respondent was asked whether they have any type of health care coverage including health insurance, prepaid plans, state or national health care plans or veteran’s benefits. Our results show that 57.9%, 43.2%, 12.8%, and 58.1% of Galveston, CEEJ, MVC, and UHN residents, respectively, have access to some sort of health care coverage (Figure 2). Although healthcare coverage was lowest for the MVC community, they reported the highest rate of ‘know of a place where they can get care.’ 

### 3.6. Seafood Consumption

**Figure 3:**
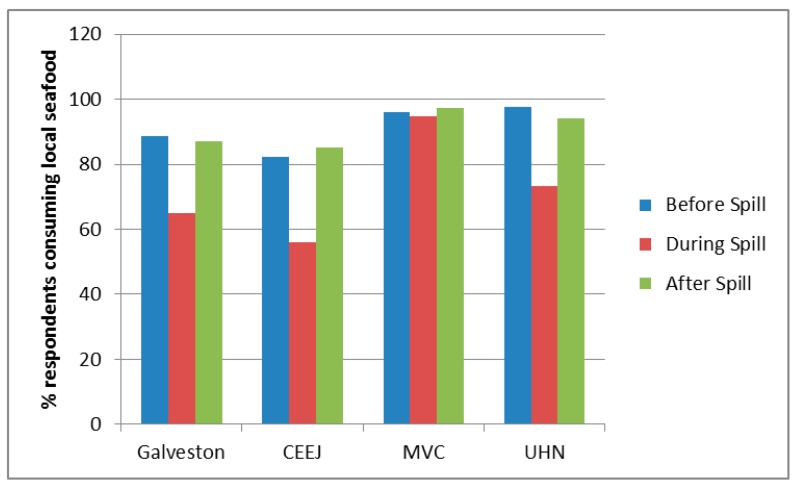

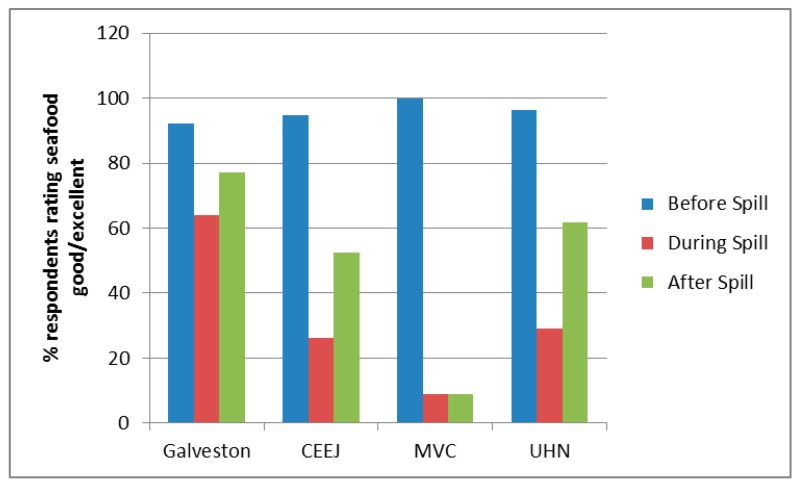
Seafood consumption and perceived quality of Gulf seafood before, during, and following spill. The percentage of respondents from each community that self-reported eating any type of seafood before (blue bars), during (red bars), and after (green bars) the oil spill (upper panel), and perceived quality of seafood before, during, and after the spill (lower panel).

We also investigated seafood consumption patterns of participants to determine if participants altered the amounts and types of Gulf seafood consumed as a result of the spill. Respondents were simply asked whether or not they consumed seafood during a particular time: before, during or after the oil spill. Overall, communities from MVC and UHN had the highest baseline seafood consumption with 96% and 98% of respondents from those communities stating they consumed seafood prior to the oil spill, respectively (Figure 3, upper panel). A majority of respondents from Galveston and CEEJ also consumed seafood prior to the spill (89% and 82% respectively). Most communities showed a decrease in seafood consumption during the oil spill with CEEJ having the largest decrease (32%) and MVC having the lowest decrease (1%). However, after the oil spill, all communities appear to recover to nearly baseline regarding the percentage of respondents reporting consuming seafood, which is surprising, given the widespread perception that seafood quality was poorer following the spill (Figure 3, lower panel). 

### 3.7. Predictors of Self-Rated Health and Post Traumatic Stress Disorder

The results of logistic regression models predicting the odds of good to very good self-rated health after the oil spill are presented in Table 2. Compared to the Galveston community, each of the other communities had reduced odds of reported good/very good health; however, after adjusting the models for covariates, these differences are no longer significant for the UHN community (odds ratio (OR) 0.46, 95% confidence level (CI) 0.18–1.18). In the final model, heart trouble was associated with lower odds of good/very good health (OR 0.23, 95% CI 0.06–0.87), as was a change in living situation due to the spill (OR 0.23, 95% CI 0.08–0.61) and direct exposure to the oil spill (OR 0.35, 95% CI 0.13–0.91). Sensitivity analyses using multiple imputation with chained equations resulted in no substantive differences in the results.

The results of the negative binomial model predicting count of PTSD symptoms are presented in Table 3. The initial model shows the strong association of increased PTSD symptoms in the CEEJ, MVC and UHN communities compared to Galveston. However, in the final, full model, these differences are not significant. High coping was associated with 0.63 symptoms (95% CI 0.42–0.96) compared to those with low coping. Changes in living situation due to the spill and direct exposure to the spill were both associated with increased symptoms of PTSD (incidence rate ratio (IRR) 1.86, 95% CI 1.18–2.93 and IRR 2.85, 95% CI 1.79–4.53 respectively). Sensitivity analyses using multiple imputation with chained equations resulted in no substantive differences in the results.

## 4. Discussion

The GC-HARMS consortium, which includes a team of researchers and community groups using CBPR methods, is conducting a human health assessment of gulf communities that may have been exposed to petrogenic PAHs following the Macondo/Deepwater Horizon oil spill. This report describes findings from a questionnaire administered by the GC-HARMS consortium, which elicited information regarding physical and mental health, resiliency and social support, economic stress, and patterns of seafood consumption. A comparison of adverse health issues and concerns reported by three communities affected by the DWH oil spill disaster (two from Mississippi and one from Louisiana) to those reported by residents living in a non-affected community (Galveston, TX, USA) was conducted. 

Standard recovery planning suggests that psychosocial recovery begins around 1 to 2 years following a catastrophic event [34]. Anxiety and depression are often elevated immediately following oil spills and can persist for months [15,16,17,18,19,27], while some research suggests symptoms can last for many years [35]. Three years following the Macondo/Deepwater Horizon spill, we found that residents of areas directly affected by the spill did not have appreciably different levels of depression (as measured by the PHQ-8) or anxiety (measured by the GAD-7) compared to those in the unaffected community (Galveston). In addition, rates of severe anxiety in these communities 3 years following the spill (1.3–2.5%) were similar to national averages (about 1%) [36], and rates of severe depressive symptoms (1.3% to 6.3%) were below reported national rates of major depressive episodes (6.7%) [37]. It is possible that any elevations in depressive symptoms and anxiety following the spill had declined by the time the survey was administered. Alternatively, it is possible that the spill itself was not a contributing factor to those particular aspects of mental health in our sample. Gould and colleagues found very little difference in levels of major depressive episodes, serious mental illness, or anxiety prior to or during the spill [38]. 

In contrast to the lack of observed difference in anxiety or depression, we did find substantial differences in the prevalence of PTSD across the communities sampled at 3 years. As expected, we found no evidence of PTSD in the Galveston community which was not exposed to the adverse effects of the spill, while increasing prevalence was found in the CEEJ, UHN and MVC communities (6%, 10% and 19% respectively). Of particular note is the high degree of direct oil spill contact reported in the MVC and UHN communities, and the large association of direct spill contact with increased levels of PTSD measures in our regression models. The lingering of PTSD symptoms is not surprising. PTSD is common following disasters and is related to exposures (e.g., exposure to traumatic event, life disruption) and mitigating factors such as social support and coping skills [39]. Limited data are available regarding the course of PTSD over time. Arata and colleagues reported elevated levels of PTSD at six years following the Exxon Valdez disaster [26]. Hansel and colleagues reported that PTSD symptoms were present at up to two years following the Macondo/Deepwater Horizon spill in their sample [16]. Their reported prevalence (21%) was higher than the levels we detected, the highest of which were in the MVC and UHN communities (19% and 10% respectively). Consistent with other research, we found that degree of exposure was associated with increases in PTSD while scoring high on the coping scale was associated with a reduction in PTSD symptoms [18,39]. The continued presence of PTSD in the GC-HARMS communities underscores the need for long-term mental health assessments and importance of access to mental health care services—particularly in the MVC community in which only 8% reported such access.

We found that self-reported health prior to and after the spill indicates movement from Very Good or Good to Fair or Poor. This may be influenced by the fact that there are marked disparities in access to health care—even in the same geographic communities. Our multivariate models also showed significant differences between the CEEJ and MVC communities compared to Galveston. Participants from the Vietnamese community report little access to health care and virtually no access to care in which trained medical interpreters are provided. While we did bring a Vietnamese-fluent practitioner as part of our study team to assist with examinations and interpretations of findings, participants reported that few such resources are available locally. In other areas, including Louisiana, resources are not readily available for more rural communities, especially for those who are increasingly isolated by persistent erosion of the coast and the intrusion of the Gulf into what was formerly habitable land. In the course of our study, many participants reported not having seen a doctor in years, despite previous diagnoses and clinical evidence of multiple chronic diseases such as diabetes, hypertension, and coronary artery disease. At the urging of our local coordinators, we identified clinical treatment facilities prior to beginning the study. This was of critical importance, given that in the course of conducting the physical examinations, our physician identified potentially life-threatening concerns, which entailed obtaining immediate medical care, with the help of our community partners. 

It is concerning that despite marked differences in perception of seafood quality prior to, during, and after the spill, most communities report relatively unchanged consumption of seafood. Possible reasons for this are many: most participants and their families involved in fishing commercially report they are frequently unable, or unwilling, to readily change their diet, regardless of their belief that the seafood quality has declined—these participants proclaim a deep dependency on the Gulf of Mexico being healthy as their livelihoods depend on such. Culture and history are also important factors as are economic factors. For example, many participants report that their daily and/or seasonal catches have declined since the spill, rendering them less economically able to afford alternate foods. 

Perceived risk in exposure and economic resource groups have comparable high levels of worry about the impact of the DWH oil spill on the environment, human health, and seafood safety [17]. Many participants in our study reported negative financial impacts resulting from the closure of the Gulf for fishing and for drilling. It is essential to recognize that coastal communities have been and are affected by many different environmental threats including hurricanes, industrial accidents and upsets, as well as coastal erosion. There is a consensus that disasters will continue in the future. This study adds to the growing body of literature which indicates that persons affected by the DWH and post-disasters are experiencing increased risks of physical health and mental health symptoms [9,10,24,27]. 

### Limitations and Future Studies

Several limitations should be considered when interpreting the results. This study used a self-report, cross-sectional approach; therefore, causality cannot be established. The lack of baseline exposure is another important study design challenge. As is common in disaster research, we did not have pre-exposure health data for our study participants and cannot unequivocally identify causal factors for the high prevalence of physical health and mental health symptoms found. There may be unmeasured factors that underlie the variables that we examined. Data collection for study participants was conducted during the following timeframe: 13 August 2013 through 12 December 2013; and for the comparison community, 27 April through 2 May 2014. However, our inclusion of a comparison group that was unaffected by the Macondo/Deepwater horizon spill allowed us to assess relative differences between groups at three years following the spill. 

When using a cross-sectional study design, there is a chance of selection bias, whereby individuals with poor health may be too ill to participate in a research study. Conversely, it is also possible that individuals with health concerns are more motivated to participate than those individuals who are not concerned about their health. In addition, study participants may be motivated to participate due to positive feelings associated with participating in a study of the health effects of the oil spill that may be of value to their community.

An additional limiting factor may be response bias, whereby participants reported increased symptoms due to being aware that they may have been exposed. Conversely, it is possible that any individuals or family members involved in oil spill clean-up did not wish to complain about health concerns or participate in the study for fear of job loss.

A central challenge in study design is the lack of baseline exposure data and population data. We attempted to alleviate this discrepancy by asking participants to recall their health symptoms prior to the spill, during the spill and on their current health after the spill. Recall Bias can be problematic in studies that rely on self-reported measure. In reporting chronic health effects, an individual can experience recall bias concerning the nature and magnitude of earlier exposures and frequency of symptoms. Using a detailed questionnaire with internal checks helps to control for this bias. 

A strength of this study is that Physicians performed rigorous health assessments that included measurements of blood pressure, heart rate, height, weight, waist circumference, visual acuity, lung function, blood, and urine analysis. An additional strength was the use of random selection and high response rates, thus reducing the chances of selection bias and overestimation of health-related symptoms. There is a lack of information in terms of health effects of susceptible groups to oil spills [10]. Notably, we included diverse community groups in our study, which may influence different exposures or health outcomes. We also used a CBPR approach, heavily involving our communities, which has contributed greatly to our ability to carry out the study. However, participants largely do not report that they trust information from industry, the government, or academia. In fact, the number one reported trusted source of information is the medical profession. This being the case, we must ensure that dissemination efforts include the health professions, ultimately leading to improved medical and health education that improves the capacity for diagnosis and treatment of environmentally induced conditions. 

## 5. Conclusions

This study adds to the growing body of information that indicates that individuals living in the communities directly affected by the Macondo oil spill have experienced an increase in the prevalence of mental health and physical health symptoms. Caution should be taken when analyzing and drawing conclusions in the context of our findings, as they may not be generalizable. Whether there is a long-term continuation of symptoms in these individuals is yet to be determined. As follow-up data become available, the GC-HARMS consortium will provide updates helping to bridge the knowledge gap on the long-term health effects of communities impacted by oil spills. Evaluating the effects of oil spills on human health is challenging but necessary. While our study areas are well defined, the outreach and engagement target population is widely diffuse, comprising residents of all affected Gulf Coast States. Clear communication of findings, not only from our study or from the work of the other U19 consortium members, but also from all ongoing studies being conducted in the Gulf of Mexico is essential to better protect the health of currently affected populations as well as those that will be impacted by future public health disasters. 

### 5.1. General Implications

Data collection in communities adversely and possibly disproportionately affected by disasters is necessary to accommodate not only post-disaster comparisons in the future but longitudinal cohort studies examining health outcome trends over time. Research on both mental and physical health parameters should be conducted as both often manifest together. We seek to not overgeneralize findings but lay the groundwork for future study related to the selected communities over time, while also adding what we discover to help lay the groundwork for CBPR approaches to post-disaster response and further study. The lack of baseline exposure is an important study design challenge in this wave 1 study, meant to allow for the acquisition of ‘baseline’ findings that provide descriptive information for a multi-wave, longitudinal observational study. Future results of our research endeavor will be strengthened via a longitudinal cohort study design that examines health outcomes over time, whereby study participants will serve as their own controls. The findings from this study will be used to compare to future waves of data collection. Collection of participant surveys, plasma and urine samples, and seafood samples will continue to be conducted and tested for petrogenic PAH levels to fulfil the overall objective of the GC-HARMS consortium which is to (1) assess seafood contamination; (2) determine PAH toxicity; (3) evaluate exposure and health outcomes in a longitudinal cohort study; and (4) disseminate findings to stakeholders.

### 5.2. Policy Implications

Policymakers should be aware that data from studies that show associations between self-reported environmental factors and health symptoms could arise from the correlation of many risk factors. Knowledge gained from this study along with other peer-reviewed studies conducted in relation to the Macondo (DWH) oil spill may have a significant impact on future public health responses to similar disasters. There is a lack of pre-disaster baseline data for many communities at risk for disaster emergencies (e.g., man-made or natural). Future funding related to disaster preparedness research should include means to work with communities to acquire pre-disaster baseline data so that before the event of future oil spills or another disaster occurs, researchers can proactively obtain and share access to this important information in order to more accurately gauge health impacts on our communities over time. Baseline data is paramount to accommodate not only post-disaster comparisons in the future but for conducting longitudinal cohort studies examining health outcome trends over time. Also important is the ability to collect biomonitoring and health data immediately after a disaster occurs through the adoption of rapid protocols. Although we did not have pre-disaster data available for this study, we would like to highlight the success of this project using the CBPR approach that depended heavily upon early and active involvement of our communities. They helped with the development of the proposal, collected samples, maintained registries and communications with participants, and disseminated results in appropriate manners and venues. Our strong belief is that for disaster research in particular, this must become the norm rather than the exception. As vital as our “first responders” are in such situations, they are rarely genuinely first. Those who live, work, and play in these vulnerable coastal communities most assuredly are. We must ensure that each community is equipped, trained, funded, and educated to respond to emergencies in their midst. We must have protocols and policies in place to rapidly take the field to prevent exposures and protect individual and public health. 

## Figures and Tables

**Table 1:** Characteristics of Gulf Coast Health Alliance: health Risks related to the Macondo Spill (GC-HARMS) sampled adults 18 years and older by interview site-mean (standard deviation; SD) or %.

	Total	Galveston	CEEJ	MVC	UHN	
*n*	324	80	79	78	87	
Age	45.7 (15.5)	45.8 (17.3)	42.7 (15.5)	49.4 (12.5)	45.2 (15.9)	
Female	56.2	55	60.8	51.3	57.5	
Ethnicity						**
Asian	25.23	1.3	2.5	100	0	
Black	38.5	46.3	83.5	0	25.3	
Hispanic	3.1	10	1.27	0	1.15	
Native American	12	0	0	0	44.8	
Other	0.6	0	1.27	0	1.15	
White	20.6	42.5	11.4	0	27.6	
BMI	30.3 (7.3)	29.7 (6.8)	32.6 (8.0)	25.7 (3.5)	32.9 (7.5)	**
Overweight	30.8	30	20.3	46.8	26.4	**
Obese	43.7	41.3	59.5	11.4	60.9	***
Smoke	28.1	18.9	26.1	20.8	44.1	**
Exercise	53.6	57.5	56.9	54.7	46.5	
Health Pre-spill (good/excellent)	69.8	74.3	58.4	75	71.3	
Health Post-spill (good/excellent)	38.7	74.2	38.6	5.3	43.2	***
Health Conditions						
Hypertension	38.5	26.3	42.2	34.3	48.8	**
Diabetes	23	12.3	22.1	24.3	32.1	**
Cancer	7.5	6.8	8.3	4.2	10.3	
Heart Trouble	10.7	8	16	6.7	11.8	
Stroke	2.9	6.6	2.7	1.3	1.2	
Anxiety	13.9	12.5	10.1	12.7	19.5	
Depressive Symptoms	13.9	12.5	13.9	12.7	16.1	
PTSD	8.9	0	6.3	19	10.3	***
Coping-Low	8.7	6.4	6.4	15.4	6.9	
Coping-High	63.2	75.6	82.1	28.2	66.7	***
Social Support (always/usually)						
Someone to Talk to	62.8	68.4	58.4	57.7	66.3	
Similar Situation	54.3	45.5	42.1	71.8	57.1	***
Received Help	19.5	25	13.16	21.8	18.1	***
Turn to Family	49.4	64.9	45.3	25.6	61.5	***
Stress Exposure						
Unemployed	33.2	5.7	32.9	71	25.6	***
Lost Income	48.8	7.1	46	83.6	56	***
Change Living Situation	31.2	5.6	29.9	61.6	27.9	***
Directly Exposed to Spill	23.2	0	15.5	52	25.6	***
Healthcare Access						
Any Kind of Coverage	43.3	57.9	43.2	12.8	58.1	***
Mental Health Coverage	33.6	43.9	34.9	8.3	53.5	***
Healthcare Professionals	52.7	59.7	52.1	26.4	70.9	***
Know of Clinic	91.5	88.6	82.4	96.1	97.6	**
Seafood Consumption (any)						
Before Spill	91.5	88.6	82.4	96.1	97.6	***
During Spill	73.2	65.1	55.9	94.9	73.2	***
After Spill	91.2	87.1	85.1	97.4	94.1	*
Seafood Quality (good/excellent)						
Before Spill	95.9	92.1	94.8	100	96.5	
During Spill	31.8	64	26.3	9	29.1	***
After Spill	50	77	52.6	9	61.9	***

* *p* ≤ 0.05, ** *p* ≤ 0.01, *** *p* ≤ 0.001 Note: means compared with ANOVA and Tukey test, differences in contingency tables assessed with Fisher’s exact test. Center for Environmental and Economic Justice (CEEJ); Mississippi Vietnamese Fishing Community (MVC); United Houma Nation (UHN); Body Mass Index (BMI); Post Traumatic Stress Disorder (PTSD).

**Table 2:** Logistic regression predicting odds of reporting good to excellent health following the oil spill among adults 18 years and older in the GC-HARMS sample.

									Imputed Dataset	
	OR	(95% CI)	OR	(95% CI)	OR	(95% CI)	OR	(95% CI)	OR	(95% CI)
CEEJ	0.22	(0.10–0.46)	0.20	(0.09–0.45)	0.19	(0.08–0.43)	0.33	(0.13–0.83)	0.26	(0.11–0.62)
MVC	0.02	(0.01–0.06)	0.02	(0.00–0.05)	0.02	(0.01–0.07)	0.06	(0.02–0.22)	0.04	(0.01–0.16)
UHN	0.27	(0.13–0.55)	0.24	(0.11–0.56)	0.26	(0.11–0.60)	0.46	(0.18–1.18)	0.41	(0.17–1.02)
Age			0.98	(0.96–1.00)	0.98	(0.96–1.00)	0.98	(0.96–1.00)	0.98	(0.96–1.00)
Female			1.03	(0.56–1.88)	0.98	(0.53–1.82)	0.88	(0.45–1.71)	0.75	(0.40–1.41)
Weight: over			2.76	(1.15–6.65)	2.55	(1.05–6.22)	2.00	(0.77–5.18)	1.99	(0.78–5.03)
Weight: Obese			1.43	(0.65–3.13)	1.37	(0.62–3.06)	1.09	(0.44–2.70)	1.14	(0.49–2.63)
Heart Trouble			0.23	(0.07–0.74)	0.24	(0.08–0.78)	0.23	(0.06–0.87)	0.26	(0.07–0.91)
Coping: High					1.95	(0.90–4.22)	1.93	(0.83–4.51)	1.56	(0.70–3.49)
Living Situation							0.23	(0.08–0.61)	0.26	(0.10–0.66)
Spill Exposure							0.35	(0.13–0.91)	0.33	(0.13–0.87)
*n*	282		272		272		250		281	

Odds Ratio (OR); Confidence Interval (CI).

**Table 3:** Negative binomial regression predicting count (IRR) of PTSD measures following the oil spill among adults 18 years and older in the GC-HARMS sample.

									Imputed Dataset	
	IRR	(95% CI)	IRR	(95% CI)	IRR	(95% CI)	IRR	(95% CI)	IRR	(95% CI)
CEEJ	2.23	(1.20–4.12)	2.35	(1.26–4.38)	2.45	(1.31–4.56)	1.11	(0.56–2.20)	1.30	(0.69–2.45)
MVC	3.54	(1.96–6.41)	3.65	(1.96–6.80)	2.85	(1.51–5.38)	0.76	(0.34–1.73)	0.96	(0.45–2.06)
UHN	2.85	(1.56–5.21)	2.89	(1.57–5.31)	2.50	(1.38–4.52)	1.06	(0.53–2.12)	1.25	(0.65–2.39)
Age			0.99	(0.98–1.00)	0.99	(0.98–1.00)	0.99	(0.97–1.00)	0.99	(0.98–1.00)
Female			0.67	(0.46–0.99)	0.69	(0.47–1.02)	0.94	(0.62–1.42)	0.91	(0.62–1.33)
Heart trouble			1.86	(1.16–2.96)	1.80	(1.14–2.84)	1.52	(0.90–2.54)	1.46	(0.92–2.31)
Coping: High					0.50	(0.33–0.74)	0.63	(0.42–0.96)	0.58	(0.39–0.86)
Unemployed							1.38	(0.87–2.21)	1.18	(0.74–1.87)
Income Loss							1.15	(0.64–2.05)	1.20	(0.68–2.12)
Living Situation							1.86	(1.18–2.93)	1.76	(1.11–2.79)
Spill Exposure							2.85	(1.79–4.53)	2.53	(1.65–3.86)
*n*	324		309		308		251		324	
